# Self-reported oral health indicators and glycemic status in community-dwelling adults: evidence from the Uonuma cohort study

**DOI:** 10.3389/froh.2026.1883455

**Published:** 2026-06-15

**Authors:** Kaung Myat Thwin, Kumiko Minagawa, Noboru Kaneko, Akihiro Yoshihara, Kana Suwama, Ayuko Odajima, Keiko Kabasawa, Yumi Ito, Masanori Iwasaki, Junta Tanaka, Hiroshi Ogawa

**Affiliations:** 1 Division of Preventive Dentistry, Faculty of Dentistry, Graduate School of Medicine, Dentistry and Health Sciences, Niigata University, Niigata, Japan; 2 Department of Health Informatics, Faculty of Healthcare Management, Niigata University of Health and Welfare, Niigata, Japan; 3 Division of Oral Science for Health Promotion, Faculty of Dentistry, Graduate School of Medicine, Dentistry and Health Sciences, Niigata University, Niigata, Japan; 4 Department of Health Promotion Medicine, Graduate School of Medicine, Dentistry and Health Sciences, Niigata University, Niigata, Japan; 5 Division of Clinical Nephrology and Rheumatology, Kidney Research Center, Graduate School of Medicine, Dentistry and Health Sciences, Niigata University, Niigata, Japan; 6 Division of Dental Public Health, Department of Oral Health Science, Graduate School of Dental Medicine, Hokkaido University, Sapporo, Japan; 7 Health and Wellness Center, University of Niigata Prefecture, Niigata, Japan

**Keywords:** diabetes, glycemic status, HbA1c, Japan, masticatory ability, periodontal disease, tooth loss

## Abstract

**Background:**

Although oral health has been linked to glycemic outcomes, its association with different stages of glycemic dysregulation remains unclear. This study examined the associations of self-reported periodontal disease history, masticatory ability, and remaining tooth count with HbA1c-based glycemic status among community-dwelling adults.

**Methods:**

This cross-sectional study used baseline data from the Uonuma CKD Cohort Study, including 4,338 participants aged ≥40 years from Niigata Prefecture, Japan. Oral health indicators included self-reported periodontal disease history, masticatory ability, and remaining tooth count. HbA1c-based glycemic status was classified as normal-range (<5.7%), prediabetes-range (5.7–6.4%), or diabetes-range (≥6.5%). Multinomial logistic regression was used to estimate relative risk ratios (RRRs) and 95% confidence intervals (CIs) for associations with glycemic category across four sequential adjustment models. Poisson regression with robust variance estimation was additionally performed for dysglycemia (HbA1c ≥ 5.7%) as a secondary outcome.

**Results:**

The median age was 66.0 years, and 53.7% were female. Prediabetes-range and diabetes-range HbA1c were observed in 33.6% and 5.9% of participants, respectively. Periodontal disease history was associated with prediabetes-range HbA1c and dysglycemia in unadjusted and partially adjusted models, but these associations were attenuated after full adjustment. Masticatory ability showed no significant association with any glycemic outcome in adjusted analyses. Edentulous participants had significantly higher RRRs of diabetes-range HbA1c compared to those with ≥20 teeth in unadjusted (RRR: 2.25, 95% CI: 1.35–3.75) and age- and sex-adjusted models [adjusted RRR (aRRR): 1.63, 95% CI: 1.17–2.75], but this association was attenuated after further adjustment.

**Conclusions:**

Self-reported periodontal disease history was most consistently associated with prediabetes-range HbA1c and dysglycemia, although these associations were attenuated after full adjustment. Tooth loss was associated with diabetes-range HbA1c before full adjustment, suggesting that shared metabolic and lifestyle-related factors may account for part of the observed attenuation. Prospective studies using clinically verified oral health assessments are needed to clarify temporal relationships and potential mechanisms underlying these associations.

## 1 Introduction

Diabetes mellitus (DM) is a major public health challenge, with an estimated 537 million adults affected worldwide in 2021 and a projected increase to more than 780 million by 2045 (1, 2). This growing burden reflects not only population growth but also increasing age-standardized prevalence, driven by factors such as sedentary lifestyles, obesity, and population aging (2, 3). In Japan, approximately 11 million adults were living with diagnosed DM as of 2024, and the prevalence has increased particularly among middle-aged and older adults, placing the country among those with the highest number of affected individuals (3, 4). As Japan's population continues to age rapidly, the burden of impaired glycemic regulation is expected to increase further (4). In addition to established DM, prediabetes has similarly increased in parallel and become an important target for prevention because it represents an intermediate and potentially reversible stage before overt diabetes (1, 5). Glycated hemoglobin (HbA1c), which reflects average blood glucose levels over the preceding two to three months, is widely used to assess the glycemic status across the spectrum from normal glycemia to prediabetes and DM (6). Therefore, identifying modifiable systemic factors associated with HbA1c-defined glycemic status may help support earlier prevention strategies in community-dwelling populations.

Among the risk factors receiving increasing research attention, oral health has emerged as a particularly compelling candidate, given the biological plausibility of a link between chronic oral inflammation and impaired glucose metabolism (7, 8). In particular, periodontal disease has received specific attention due to its proposed bidirectional association with DM, mediated through shared inflammatory and metabolic pathways (8). Tooth loss and impaired masticatory function may also be associated with glycemic status through reduced occlusal support, altered dietary intake, and nutritional imbalance (9–14). Importantly, these oral health conditions are not entirely independent: periodontal disease is a major cause of tooth loss, and reduced tooth count may impair masticatory function (15–17). This interrelationship supports examining these indicators together as related but distinct inflammatory, structural, and functional dimensions of oral health, rather than as completely isolated exposures. Previous studies have reported inverse associations between number of remaining teeth or masticatory performance and both prevalence of DM and HbA1c levels (10, 11, 13). Moreover, impaired chewing ability was independently linked with elevated risks of DM, regardless of other risk factors (9).

Despite accumulating evidence linking oral health to glycemic outcomes, several gaps remain. First, most prior studies have examined periodontal disease, tooth loss, and masticatory function as isolated exposures, and population-level evidence comparing all three indicators within the same community sample remains limited. Second, many previous studies have focused on individuals with established diabetes, whereas less is known about oral health indicators across earlier stages of glycemic dysregulation, including prediabetes-range HbA1c. Third, although studies in community-dwelling adults have reported associations of periodontal status and tooth loss with diabetes, evidence from community-dwelling Japanese adults remains limited, particularly for analyses comparing multiple self-reported oral health indicators across HbA1c-defined glycemic categories (18, 19). Therefore, this study examined the associations of self-reported periodontal disease history, masticatory ability, and remaining tooth count with HbA1c-based glycemic status, across three categories, among community-dwelling adults using baseline data from the Uonuma CKD Cohort Study. By evaluating these three oral health dimensions within the same population sample and examining outcomes across the full glycemic spectrum, this study may provide evidence relevant to understanding oral-systemic health associations in community-based settings.

## 2 Materials and methods

### 2.1 Study design and participants

This cross-sectional study is the secondary analysis of baseline data from the Uonuma CKD Cohort Study, a population-based prospective cohort of community-dwelling adults aged 40 years and older in the Uonuma area of Niigata Prefecture, Japan (20). Full details of the cohort design and baseline characteristics have been described previously (20–22). Baseline questionnaire surveys and health check-up examinations were conducted between 2012 and 2015. The present study linked participants’ self-administered questionnaire data with blood test results obtained at health check-up examinations.

**Figure 1:**
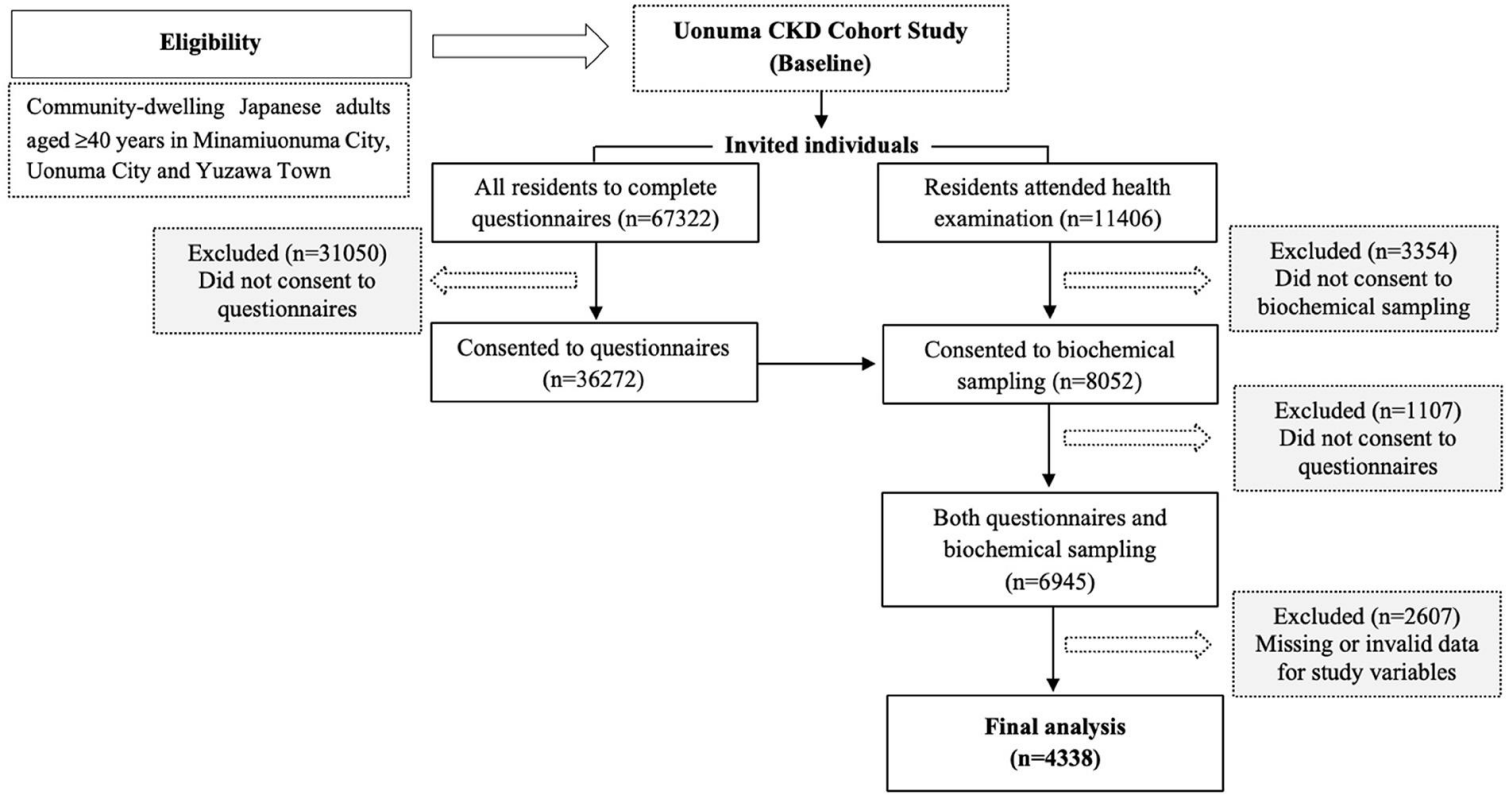
Flowchart of the study participants.

Of the 6,945 individuals who received both self-administered questionnaire and biochemical sampling, 2,607 were excluded due to missing values in any of the exposure variables, outcome variables, or covariates. Finally, the analytic dataset included 4,338 participants (Figure 1). Ethics approval was granted by the Ethics Committee of Niigata University (Approval number: 2017-0071). All procedures were conducted in accordance with the Declaration of Helsinki and all participants provided written informed consent.

### 2.2 Exposure variables

The key exposure variables used in the present study are history of periodontal disease, masticatory ability, and remaining tooth count. A history of periodontal disease was assessed by the questionnaire item: “Have you ever been diagnosed with periodontal disease by a dentist?”, with the response yes or no (reference category). This self-reported measure of periodontal disease history has been used in previous analyses of the Uonuma CKD Cohort Study, including our prior publication (21). Masticatory ability was determined from participants’ responses to the question: “Can you bite with your molars on both sides, using your own teeth or dentures?” Responses were categorized as: both sides (reference category), one side only, or neither side. Self-reported remaining tooth count was stratified into four groups: ≥20 natural teeth (reference category), 10–19 teeth, 1–9 teeth, and 0 (edentulous) (22).

### 2.3 Outcome variables

HbA1c was measured using blood samples collected during health check-up examinations. For the primary analysis, participants were classified into three categories: normal-range HbA1c (<5.7%; reference category), prediabetes-range HbA1c (5.7%–6.4%), and diabetes-range HbA1c (≥6.5%), in accordance with the diagnostic criteria of the Japan Diabetes Society (23). For the secondary analysis, dysglycemia was defined as HbA1c ≥ 5.7% (24), encompassing both prediabetes-range and diabetes-range HbA1c, to capture the broader spectrum of impaired glycemic regulation in the community-dwelling population.

### 2.4 Covariates

Covariates were selected based on their plausible confounding roles in the associations between self-reported oral health indicators and glycemic outcomes. Sociodemographic information included age (years) and sex (male or female). Waist circumference (cm) was measured at health check-up examinations and used as the primary obesity indicator (25), as body mass index data were unavailable for this cohort. Lifestyle-related factors included current smoking (yes or no), alcohol consumption (yes or no), and regular physical exercise (yes or no), which have been associated with diabetes-related metabolic outcomes in previous studies (25–28). Clinical factors included antidiabetic medication use (yes or no), antihypertensive medication use (yes or no), lipid-lowering medication use (yes or no), history of cardiovascular disease (yes or no), and urine albumin-to-creatinine ratio (uACR, mg/g). Medication use, cardiovascular disease history, and uACR were included to account for underlying cardiometabolic and renal conditions that may influence HbA1c levels or reflect shared systemic risk in the oral health–glycemic status association (29, 30).

### 2.5 Statistical analysis

Descriptive statistics for categorical variables are reported as frequencies and percentages, and for continuous variables as median (interquartile range; IQR). Normality of continuous variables was assessed using the Kolmogorov–Smirnov test. Differences in participant characteristics across three glycemic categories were assessed using the Chi-square test or Kruskal–Wallis test. For the primary analysis, multinomial logistic regression was used to estimate relative risk ratios (RRRs) and 95% confidence intervals (CIs) for the associations between self-reported oral health indicators and HbA1c-based glycemic status. Prediabetes-range HbA1c and diabetes-range HbA1c were each compared with normal-range HbA1c as the reference category. Four sequential adjustment models were applied: Model 1 (unadjusted); Model 2 (adjusted for age and sex); Model 3 (additionally adjusted for waist circumference and lifestyle-related factors); and Model 4 (fully adjusted by additionally including clinical factors). For the secondary analysis, Poisson regression with robust variance estimation was used to estimate prevalence ratios (PRs) and 95% CIs for the associations of self-reported oral health indicators with HbA1c-based dysglycemia, using the same four-model adjustment sequence. A unified complete-case sample of 4,338 participants was used throughout the analyses to ensure comparability across models. Statistical significance was set at a two-tailed *p*-value of <0.05. All analyses were performed using SPSS version 27.0 for Windows (IBM Corp., Armonk, NY, USA).

## 3 Results

Among the 4,338 participants included in the analysis, the median age was 66.0 (IQR: 60.0–71.0) years, and 46.3% were male. Of these, 1,459 (33.6%) had prediabetes-range HbA1c and 257 (5.9%) had diabetes-range HbA1c levels. The overall median HbA1c value was 5.66 (IQR: 5.40–5.90). Participants in higher glycemic categories were significantly older (*p* < 0.001) and those with diabetes-range HbA1c were more likely to be male (*p* < 0.001) (Table 1). Significant differences across glycemic groups were observed in waist circumference, lifestyle-related factors, medication use, cardiovascular disease history, and uACR. The history of periodontal disease (*p* = 0.034) and the distribution of remaining tooth count (*p* = 0.002) differed significantly across glycemic categories, whereas the distribution of masticatory ability did not differ significantly.

**Table 1:** Characteristics of study participants according to HbA1c-based glycemic status (*n* = 4,338).

Variable	Total (*n* = 4338)	Normal-range HbA1c (*n* = 2622)	Prediabetes-range HbA1c (*n* = 1459)	Diabetes-range HbA1c (*n* = 257)	*p*-value
Age, years	66.0 (60.0–71.0)	65.0 (59.0–70.0)	67.0 (63.0–71.0)	68.0 (65.0–72.0)	**<0**.**001**
Male sex	2010 (46.3)	1219 (46.5)	615 (42.2)	161 (62.6)	**<0**.**001**
Waist circumference, cm	81.5 (76.0–87.0)	80.2 (74.8–85.8)	82.8 (77.3–88.1)	86.4 (82.0–92.7)	**<0**.**001**
Current smoking	719 (16.6)	469 (17.9)	198 (13.6)	52 (20.2)	**0**.**001**
Alcohol drinking	2399 (55.3)	1543 (58.8)	719 (49.3)	137 (53.3)	**<0**.**001**
Regular exercise	1531 (35.3)	886 (33.8)	530 (36.3)	115 (44.7)	**0**.**001**
Antidiabetic medication	222 (5.1)	14 (0.5)	82 (5.6)	126 (49.0)	**<0**.**001**
Antihypertensive medication	1260 (29.0)	657 (25.1)	482 (33.0)	121 (47.1)	**<0**.**001**
Lipid-lowering medication	872 (20.1)	372 (14.2)	409 (28.0)	91 (35.4)	**<0**.**001**
Cardiovascular disease history	168 (3.9)	70 (2.7)	77 (5.3)	21 (8.2)	**<0**.**001**
uACR (mg/g)	10.0 (6.0–20.0)	9.0 (5.0–18.0)	11.0 (6.0–22.0)	18.0 (8.8–45.0)	**<0**.**001**
Periodontal disease (yes)	1941 (44.7)	1132 (43.2)	685 (46.9)	124 (48.2)	**0**.**034**
Masticatory ability					
Both sides	3365 (77.6)	2058 (78.5)	1110 (76.1)	197 (76.7)	0.071
One side only	631 (14.5)	367 (14.0)	233 (16.0)	31 (12.1)	
Neither side	342 (7.9)	197 (7.5)	116 (8.0)	29 (11.3)	
Remaining tooth count					
≥20 teeth	2956 (68.1)	1828 (69.7)	974 (66.8)	154 (59.9)	**0**.**002**
10–19 teeth	788 (18.2)	461 (17.6)	277 (19.0)	50 (19.5)	
1–9 teeth	437 (10.1)	243 (9.3)	160 (11.0)	34 (13.2)	
0 teeth (edentulous)	157 (3.6)	90 (3.4)	48 (3.2)	19 (7.4)	

Values are presented as median (IQR) or n (%). uACR, urine albumin-creatinine ratio; Normal-range HbA1c, <5.7%; Prediabetes-range HbA1c, 5.7%–6.4%; Diabetes-range HbA1c, ≥6.5%. Variables are analyzed by Kruskal–Wallis test or Chi-square test. Bold values indicate statistical significance (*p* < 0.05).

Table 2 presents the associations between self-reported oral health indicators and HbA1c-based glycemic status using multinomial logistic regression analyses. Compared with participants with normal-range HbA1c, a self-reported history of periodontal disease was significantly associated with prediabetes-range HbA1c in the unadjusted model (RRR: 1.16, 95% CI: 1.02–1.32) and remained significant after adjustment for age, sex, waist circumference, and lifestyle-related factors in Models 2 and 3. However, this association was attenuated and became non-significant in the fully adjusted model. In contrast, masticatory ability and remaining tooth count were not significantly associated with prediabetes-range HbA1c in any model.

**Table 2:** Multinomial logistic regression analysis on self-reported oral health indicators with HbA1c-based glycemic status.

Exposure	Model 1 RRR (95% CI)	*p*-value	Model 2 aRRR (95% CI)	*p*-value	Model 3 aRRR (95% CI)	*p*-value	Model 4 aRRR (95% CI)	*p*-value
**Prediabetes-range HbA1c vs normal-range HbA1c (reference)**								
Periodontal disease (yes)	1.16 (1.02–1.32)	**0.020**	1.14 (1.00–1.30)	**0.049**	1.16 (1.02–1.32)	**0.022**	1.12 (0.98–1.28)	0.099
Masticatory ability								
Both sides	Reference		Reference		Reference		Reference	
One side only	1.17 (0.98–1.40)	0.088	1.14 (0.95–1.36)	0.160	1.10 (0.92–1.31)	0.290	1.12 (0.93–1.34)	0.248
Neither side	1.06 (0.84–1.35)	0.615	1.03 (0.81–1.32)	0.791	1.01 (0.78–1.32)	0.927	0.97 (0.75–1.25)	0.812
Remaining tooth count								
≥20 teeth	Reference		Reference		Reference		Reference	
10–19 teeth	1.10 (0.93–1.30)	0.264	0.96 (0.81–1.14)	0.646	0.94 (0.79–1.12)	0.480	0.94 (0.79–1.12)	0.473
1–9 teeth	1.21 (0.98–1.49)	0.082	1.01 (0.82–1.25)	0.918	0.96 (0.77–1.20)	0.725	0.95 (0.76–1.18)	0.629
0 teeth (edentulous)	0.96 (0.67–1.37)	0.809	0.82 (0.57–1.18)	0.288	0.78 (0.54–1.12)	0.182	0.83 (0.57–1.20)	0.314
**Diabetes-range HbA1c vs normal-range HbA1c (reference)**								
Periodontal disease (yes)	1.23 (0.95–1.59)	0.118	1.13 (0.87–1.46)	0.361	1.23 (0.95–1.59)	0.117	1.13 (0.80–1.60)	0.496
Masticatory ability								
Both sides	Reference		Reference		Reference		Reference	
One side only	0.84 (0.57–1.25)	0.392	0.81 (0.54–1.20)	0.284	0.87 (0.59–1.28)	0.474	0.86 (0.51–1.44)	0.563
Neither side	1.57 (1.04–2.37)	**0.033**	1.31 (0.86–1.99)	0.206	1.22 (0.77–1.92)	0.395	0.65 (0.33–1.26)	0.198
Remaining tooth count								
≥20 teeth	Reference		Reference		Reference		Reference	
10–19 teeth	1.13 (0.82–1.57)	0.453	0.96 (0.69–1.34)	0.818	0.94 (0.66–1.33)	0.715	0.73 (0.46–1.15)	0.175
1–9 teeth	1.49 (1.02–2.19)	**0.041**	1.15 (0.78–1.70)	0.477	1.07 (0.71–1.63)	0.735	0.79 (0.44–1.41)	0.422
0 teeth (edentulous)	2.25 (1.35–3.75)	**0.002**	1.63 (1.17–2.75)	**0.008**	1.42 (0.82–2.48)	0.213	1.23 (0.58–2.62)	0.590

RRR, relative risk ratio; CI, confidence interval; aRRR, adjusted relative risk ratio. Normal-range HbA1c (reference), <5.7%; Prediabetes-range HbA1c, 5.7%–6.4%; Diabetes-range HbA1c, ≥6.5%. Model 1: unadjusted model; Model 2: adjusted for age and sex; Model 3: adjusted for age, sex, waist circumference, current smoking, alcohol drinking, and regular exercise; Model 4: adjusted for age, sex, waist circumference, current smoking, alcohol drinking, regular exercise, antidiabetic medication, antihypertensive medication, lipid-lowering medication, cardiovascular disease history, and uACR. Bold values indicate statistical significance (*p* < 0.05).

**Figure 2:**
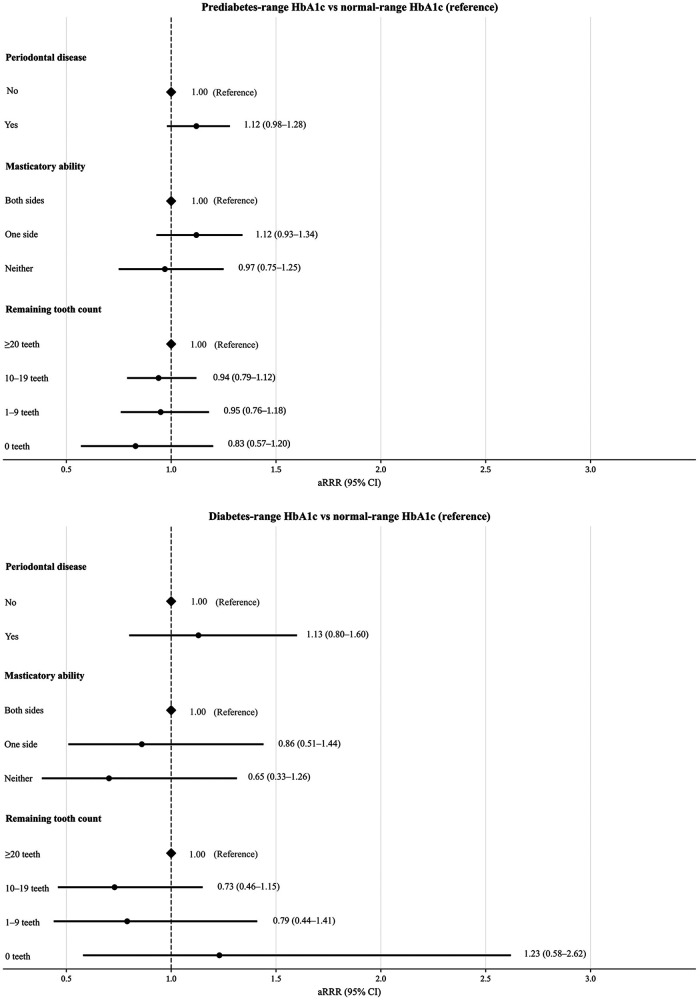
Fully adjusted associations between self-reported oral health indicators and HbA1c-based glycemic status. Values are adjusted relative risk ratios (aRRRs) and 95% confidence intervals (CI) from Model 4. Model 4 was adjusted for age, sex, waist circumference, current smoking, alcohol drinking, regular exercise, antidiabetic medication use, antihypertensive medication use, lipid-lowering medication use, cardiovascular disease history, and urine albumin-to-creatinine ratio.

For diabetes-range HbA1c, self-reported periodontal disease was not significantly associated with the outcome across all models (Table 2). Participants who reported being unable to chew on either side had a significantly higher RRR for diabetes-range HbA1c than those who could chew on both sides in the unadjusted model (RRR: 1.57, 95% CI: 1.04–2.37), but this association did not persist after covariate adjustment. Similarly, participants with 1–9 remaining teeth and edentulous participants showed significantly higher relative risks for diabetes-range HbA1c in the unadjusted model compared with those with ≥20 teeth. After adjustment for age and sex, the association among edentulous participants remained significant [adjusted RRR (aRRR): 1.63, 95% CI: 1.17–2.75], but it was attenuated after further adjustment in Models 3 and 4. The fully adjusted associations between self-reported oral health indicators and HbA1c-based glycemic status are illustrated in Figure 2.

A self-reported history of periodontal disease was significantly associated with a higher prevalence of dysglycemia, defined as HbA1c ≥ 5.7%, in the unadjusted model (PR: 1.10, 95% CI: 1.02–1.19), after adjustment for age and sex (aPR: 1.08, 95% CI: 1.01–1.16), and after further adjustment for lifestyle-related factors (aPR: 1.09, 95% CI: 1.02–1.17) (Supplementary Table 1). However, this association was attenuated and became non-significant in the fully adjusted model. No significant associations were observed between masticatory ability and dysglycemia across any model. For remaining tooth count, participants with 1–9 teeth showed a significantly higher prevalence of dysglycemia in the unadjusted model compared with those with ≥20 teeth (PR: 1.14, 95% CI: 1.02–1.27), but this association was no longer significant after adjustment for additional covariates.

## 4 Discussion

This cross-sectional analysis investigated the associations between self-reported oral health indicators and HbA1c-based glycemic status among community-dwelling Japanese adults. The principal finding was that self-reported periodontal disease history was significantly associated with prediabetes-range HbA1c across Models 1 through 3, and with dysglycemia across the same adjustment levels. However, both associations were attenuated in the fully adjusted model (Model 4). Periodontal disease was not significantly associated with diabetes-range HbA1c in any model. Self-reported masticatory ability showed no significant association with prediabetes-range or diabetes-range HbA1c in the adjusted models. Self-reported remaining tooth count demonstrated associations with diabetes-range HbA1c in the unadjusted and age- and sex-adjusted models, particularly for the edentulous category, but these were substantially attenuated after further adjustment for lifestyle-related and clinical factors. Overall, these findings suggest that history of periodontal disease shows the most consistent association with impaired glycemic regulation, particularly across the prediabetes-range spectrum, though the attenuation observed after full adjustment warrants cautious interpretation.

The observed associations of periodontal disease history with prediabetes-range HbA1c and dysglycemia are consistent with previous evidence linking periodontal inflammation to impaired glycemic regulation (5, 8, 11–14). Individuals with periodontitis have been shown to have higher HbA1c levels and an increased risk of developing diabetes relative to periodontally healthy individuals (31). From a biological standpoint, periodontal inflammation may promote systemic dissemination of pro-inflammatory mediators, including interleukin-6 and tumor necrosis factor-α, which may be associated with impaired glucose metabolism (32). These mechanisms are likely relevant not only to established DM but also to earlier metabolic disturbances, which may be one possible reason why the present associations were more apparent for prediabetes-range HbA1c and the broader dysglycemia outcome than for diabetes-range HbA1c. The attenuation observed in the fully adjusted model, which additionally included antidiabetic medication use and uACR, warrants careful interpretation. In particular, antidiabetic medication use may complicate interpretation of HbA1c-defined diabetes-range status among treated participants, thereby reducing the ability to detect associations at the diabetes-range threshold (33). Nevertheless, the association of self-reported periodontal disease history with prediabetes-range HbA1c and dysglycemia in unadjusted and partially adjusted models suggests that periodontal disease history may be a relevant oral health marker in community-based glycemic risk assessment. Because these associations were attenuated after additional adjustment for clinical factors, prospective studies using clinically assessed periodontal status and repeated glycemic measurements are needed to clarify the temporal sequence and potential mechanisms underlying these associations.

In contrast, self-reported masticatory ability was not associated with either glycemic outcome in any adjusted models, which is not consistent with prior reports linking impaired masticatory function to higher diabetes risk (9, 34). The unadjusted association between being unable to chew on both sides and diabetes-range HbA1c did not persist after adjustment for potential confounding factors, suggesting that this association may have been largely explained by demographic and clinical differences rather than reflecting an independent association of masticatory function. A plausible explanation for the overall null finding is that the present study assessed masticatory ability using a single self-reported item regarding bilateral molar contact, which may not capture subtle differences in chewing efficiency that objective masticatory performance tests can detect. Moreover, the questionnaire allowed participants to report chewing ability with dentures, which may functionally compensate for tooth loss and therefore attenuate the relationship between chewing ability and metabolic outcomes.

Participants with fewer remaining teeth, particularly those with 1–9 teeth and edentulous participants, had substantially higher RRRs for diabetes-range HbA1c compared with those with ≥20 teeth in the unadjusted analyses, a finding consistent with prior literature (10, 35, 36). Although the association remained statistically significant among edentulous participants after adjustment for age and sex, it was attenuated after further adjustment for waist circumference, lifestyle-related and clinical factors. This suggests that the association between tooth loss and glycemic status may be partly explained by shared metabolic and lifestyle-related factors, rather than reflecting a direct independent association. Longitudinal studies with appropriate mediation analyses would be necessary to clarify these pathways.

This study has several strengths. The use of a large community-based cohort with objectively measured HbA1c values from health examinations reduces potential outcome misclassification. The evaluation of three self-reported oral health indicators within the same population sample allows a broader characterization of oral health status than studies focusing on a single indicator. The use of multinomial logistic regression to distinguish prediabetes-range and diabetes-range HbA1c, rather than combining impaired glycemic status into a single binary outcome, enabled a more nuanced assessment of associations across the glycemic spectrum. The inclusion of dysglycemia as a secondary outcome further captures earlier stages of metabolic impairment that are particularly relevant for prevention. Finally, the use of four sequential adjustment models allowed the stability of each association and the potential influence of confounding to be systematically evaluated.

However, several limitations should also be acknowledged. First, the cross-sectional design precludes conclusions about causality, and the temporal relationship between oral health conditions and glycemic status cannot be determined. Second, oral health indicators were self-reported rather than clinically assessed, which may introduce recall bias and misclassification. In particular, the questionnaire-based tooth count variable lacks the precision of clinical dental examination. Similarly, the masticatory ability assessed via a single item on bilateral molar contact may not fully capture chewing efficiency as measured by objective performance tests. Third, as each oral health indicator was examined in separate regression models without mutual adjustment, the independent contribution of each indicator cannot be determined and the possibility of residual correlation among the three exposures cannot be excluded. Fourth, residual confounding from factors not measured in the survey, such as dietary intake and dental care utilization, cannot be excluded. Fifth, the complete-case analytic approach reduced the sample size, and selection bias may have occurred if participants with missing data differed systematically from those included in the analysis. Finally, the geographic restriction to municipalities in Niigata Prefecture limits the generalizability of findings to the broader Japanese population.

## 5 Conclusion

Self-reported periodontal disease history was associated with prediabetes-range HbA1c and dysglycemia in unadjusted and partially adjusted models, suggesting that it may be a relevant oral health marker of prediabetes-range HbA1c and dysglycemia. However, attenuation after full adjustment indicates that shared metabolic and clinical factors may account for part of the observed association. Tooth loss was associated with diabetes-range HbA1c before adjustment for lifestyle-related and clinical factors, whereas self-reported masticatory ability showed no consistent independent association with glycemic outcomes. Future prospective studies incorporating clinically verified oral health assessments and repeated metabolic measurements are needed to clarify the temporal relationships and potential mechanisms underlying these associations.

## Data Availability

The raw data supporting the conclusions of this article will be made available by the authors, without undue reservation.
