# Supplementary material for: Self-reported oral health indicators and glycemic status in community-dwelling adults: evidence from the Uonuma cohort study
Source: Front Oral Health. 2026 Jun 15;7:1883455. doi: 10.3389/froh.2026.1883455 (PMC13310979; doi:10.3389/froh.2026.1883455)
Supplement: Supplementary file 1 [file Table1.docx]

Supplementary Table 1. Poisson regression analysis of self-reported oral health indicators associated with HbA1c-based dysglycemia

| **Exposure** | **Model 1**  **PR (95% CI)** | ***p*-value** | **Model 2**  **aPR (95% CI)** | ***p*-value** | **Model 3**  **aPR (95% CI)** | ***p*-value** | **Model 4**  **aPR (95% CI)** | ***p*-value** |
| --- | --- | --- | --- | --- | --- | --- | --- | --- |
| Periodontal disease (yes) | 1.10 (1.02–1.19) | **0.010** | 1.08 (1.01–1.16) | **0.034** | 1.09 (1.02–1.17) | **0.011** | 1.07 (0.99–1.14) | 0.075 |
| Masticatory ability  Both sides  One side only  Neither side | Reference  1.07 (0.97–1.18)  1.08 (0.95–1.23) | 0.198  0.253 | Reference  1.05 (0.95–1.17)  1.05 (0.92–1.20) | 0.292  0.465 | Reference  1.04 (0.95–1.15)  1.03 (0.90–1.18) | 0.410  0.683 | Reference  1.05 (0.95–1.15)  0.97 (0.86–1.10) | 0.332  0.684 |
| Remaining tooth count  ≥20 teeth  10-19 teeth  1-9 teeth  0 teeth (edentulous) | Reference  1.06 (0.97–1.16)  1.14 (1.02–1.27)  1.08 (0.90–1.30) | 0.213  **0.024**  0.404 | Reference  0.98 (0.89–1.08)  1.01 (0.91–1.13)  0.97 (0.81–1.17) | 0.682  0.817  0.768 | Reference  0.96 (0.88–1.06)  0.98 (0.88–1.10)  0.93 (0.77–1.12) | 0.428  0.783  0.440 | Reference  0.97 (0.89–1.06)  0.97 (0.87–1.08)  0.92 (0.77–1.10) | 0.520  0.553  0.373 |

PR: prevalence ratio; CI: confidence interval; aPR: adjusted prevalence ratio. Dysglycemia defined as HbA1c ≥5.7%.

Model 1: unadjusted model; Model 2: adjusted for age and sex; Model 3: adjusted for age, sex, waist circumference, current smoking, alcohol drinking, and regular exercise; Model 4: adjusted for age, sex, waist circumference, current smoking, alcohol drinking, regular exercise, antidiabetic medication, antihypertensive medication, lipid-lowering medication, cardiovascular disease history, and uACR. Bold values indicate statistical significance (*p*<0.05).
